# Prevalence of SARS-CoV-2 in newborns born to SARS-CoV-2-positive mothers at 2 weeks of life

**DOI:** 10.3389/fped.2024.1381104

**Published:** 2024-04-25

**Authors:** Sophia Jan, Robert Katz, David Fagan, Telmo Santos, Joanna C. Beachy, Caren Steinway, Jack Chen, Alina Tsouristakis, Briana Mancenido, Christy Leung, Emily Moore, Estelle Wilson, Lorna Lin, Michael Harte, Michelle Katzow, Lorry G. Rubin

**Affiliations:** ^1^ Department of Pediatrics, Northwell Health, New Hyde Park, NY, United States; ^2^ Department of Pediatrics, Cohen Children’s Medical Center, Queens, NY, United States; ^3^ Institute for Health System Science, Feinstein Institutes for Medical Research, Northwell Health, Manhasset, NY, United States; ^4^ Department of Pediatrics, Zucker School of Medicine at Hofstra/Northwell, Hempstead, NY, United States; ^5^ Division of Neonatology (NICU), Department of Pediatrics, Baystate Health, Springfield, MA, United States; ^6^ Department of Pediatrics Infectious Disease, Cohen Children’s Medical Center, New Hyde Park, NY, United States

**Keywords:** SARS-CoV-2, newborns, COVID-19, transmission risk, pediatric medicine

## Abstract

**Introduction:**

Limited evidence exists on management recommendations for neonates born to SARS-CoV-2-positive mothers. This study looked at transmission risk of neonates presenting for primary care in a large regional health system within New York during the early months of the COVID-19 pandemic.

**Methods:**

This was a prospective, observational study of newborns born to SARS-CoV-2-positive mothers presenting at any of the 19 Northwell Health-Cohen Children's Medical Center primary care practices who underwent another oropharyngeal/nasopharyngeal swab for detection of SARS-CoV-2 by day of life (DOL) 14.

**Results:**

Among 293 newborns born to SARS-CoV-2-positive mothers who were negative at birth, 222 were retested at DOL 14, corresponding to times with different predominant strains. Of these, seven tested positive but had no symptoms.

**Conclusion:**

The overall low transmission rates and absence of symptomatic infection support the safety of direct breastfeeding after hospital discharge with appropriate hand and breast hygiene.

## Introduction

Since the onset of the COVID-19 pandemic, continuous research into the infection of neonates born to SARS-CoV-2-positive mothers has led to updated management recommendations from both the Centers of Disease Control and Prevention and the American Academy of Pediatrics (AAP) (1, 2). While data from recent studies have shown that the highest risk of SARS-CoV-2 infection in neonates occurs when a mother is infected near the time of delivery (3), intrauterine, intrapartum, and peripartum transmission of SARS-CoV-2 from positive mothers is still rare (4–7). Results from these studies showed that the overall prevalence of babies infected immediately after birth was low, less than 3% (4–8), and few confirmed mother-to-child transmission (5–7). The objective of this study was to describe the clinical characteristics and estimate SARS-CoV-2 transmission risk to neonates presenting for primary care in a large regional health system in the New York metropolitan region in the first 5 months of the COVID-19 pandemic in the United States.

## Methods

All women admitted to labor and delivery across all Northwell Health delivery hospitals were required to undergo a nasopharyngeal swab for detection of SARS-CoV-2 by nucleic acid amplification (NAA) before delivery within 24 h of admission, regardless of maternal symptoms. All mothers positive for SARS-CoV-2 were required to wear surgical masks. Infants born to asymptomatic SARS-CoV-2-positive mothers were allowed skin-to-skin contact, direct breastfeeding, and rooming with their mothers immediately after delivery and throughout the nursery course; those born to symptomatic SARS-CoV-2-positive mothers were not. Symptomatic mothers were encouraged to express breast milk while another person fed their infants. Newborns were tested for SARS-CoV-2 by NAA via a combined oropharyngeal/nasopharyngeal swab by 24 h of life. At discharge, all mothers were encouraged to feed directly at the breast after performing hand and breast hygiene, to continue wearing masks, and were shown how to properly sanitize breast pumps. This education was reinforced at primary care visits.

Study participants included newborns born to SARS-CoV-2-positive mothers presenting at any of the 19 Northwell Health-Cohen Children's Medical Center (CCMC) primary care practices who underwent another oropharyngeal/nasopharyngeal swab for detection of SARS-CoV-2 by day of life (DOL) 14. We collected maternal, newborn, and delivery characteristics, as well as emergency room and hospitalization visits in the first 14 days of life (Table 1). This study was considered exempt by the Northwell Health IRB.

**Table 1:** Characteristics of newborns born to SARS-CoV-2-positive mothers: mothers, delivery circumstances, and home circumstances.

	Total (*N* = 222)	SARS-CoV-2 negative (*N* = 215)	SARS-CoV-2 positive (*N* = 7)	*P*-value
Maternal characteristics^a^				* *
Mean age (range)	29.6 ± 5.9 (15–45)	29.5 ± 6.0	31.7 ± 4.3	0.332
Gravid mean (range)	2.4 ± 1.7 (1–11)	2.3 ± 1.7	4.5 ± 2.1	0.001
Parity mean (range)	1.1 ± 1.2 (0–4)	1.0 ± 1.1	2.1 ± 1.2	0.010
Ever symptomatic for COVID-19				
Yes, *n* (%)	56 (25.2)	53 (94.6)	3 (5.4)	0.258
No, *n* (%)	135 (60.8)	132 (97.8)	3 (2.2)	
Unknown/missing, *n* (%)	31 (14.0)			
Newborn characteristics^b^				
Gestational age at birth in weeks, mean (range)	38.5 ± 1.6 (33–42)	38.6 ± 1.6	36.5 ± 2.3	0.001
Sex				
Female, *N* (%)	94 (42.3)	93 (98.9)	1 (1.1)	0.127
Male, *N* (%)	128 (57.7)	122 (95.3)	6 (4.7)	
Race				
White	53 (23.9)	51 (96.2)	2 (3.8)	0.996
Black/African American	67 (30.2)	65 (97.0)	2 (3.0)	
Asian	31 (14.0)	30 (96.8)	1 (3.2)	
Other	62 (27.9)	60 (96.8)	2 (3.2)	
Ethnicity				
Hispanic	41 (18.5)	38 (92.7)	3 (7.3)	0.150
Non-Hispanic	123 (55.4)	120 (97.6)	3 (2.4)	
Insurance				
Commercial, *N* (%)	68 (30.6)	68 (100)	0	0.159
Medicaid, *N* (%)	136 (61.3)	130 (95.6)	6 (4.4)	
Uninsured, *N* (%)	13 (5.9)	13 (100)	0	
Delivery method				
Vaginal, *n* (%)	156 (70.3)	153 (98.1)	3 (1.9)	0.107
Cesarean section, *n* (%)	66 (29.7)	62 (93.9)	4 (6.1)	
Birthweight in kg, mean (range)	3.1 ± 0.5 (1.54–4.4)	3.1 ± 0.5	2.7 ± 0.5	0.018
Delivery characteristics^c^				
Birth Hospital				
Long Island Jewish	140 (65.1)	138 (66.0)	2 (33.3)	0.112
North Shore University Hospital	36 (16.7)	34 (16.3)	2 (33.3)	
South Shore University Hospital	13 (6.0)	11 (5.3)	2 (33.3)	
Lenox Hill	5 (2.3)	5 (2.4)	0	
Forest Hills	2 (0.9)	2 (1.0)	0	
Huntington Hospital	3 (1.4)	3 (1.4)	0	
Non-Northwell Hospitals^d^	16 (7.4)	16 (7.7)	0	
APGAR				
1 min	8.4 ± 1.1 (1–9)	8.4 ± 1.1	8.4 ± 0.5	0.932
5 min	8.8 ± 0.6 (3–9)	8.8 ± 0.6	8.9 ± 0.4	0.907
Hospital course				
Well baby nursery	59 (70.2)	59 (100)	0	.086
NICU	25 (29.8)	23 (92.0)	2 (8.0)	
Discharged by 24 h				
Yes	87 (39.2)	84 (96.6)	3 (3.4)	0.879
No	135 (60.9)	126 (96.9)	4 (3.1)	
Precautions taken at home by DOL 14^e^				
Direct breastfeeding	122 (55.0)	117 (95.9)	5 (4.1)	0.373
Expressed breast milk	52 (23.4)	48 (92.3)	4 (7.7)	0.032
Formula	184 (82.9)	180 (97.8)	4 (2.2)	0.066

^a^ Age, gravidy: *n* = 220; parity: *n* = 215.

^b^ Gestational age: *n* = 220; race: *n* = 213; ethnicity: *n* = 164; insurance: *n* = 217.

^c^ APGAR: *N* = 213; discharged by 24 h: *n* = 217.

^d^ Non-Northwell Health Hospitals included Good Sam, St. Charles, St. Catherine's, Mt. Sinai, and Mercy.

^e^ Babies could have used a combination of feeding modalities.

## Results

**Figure 1:**
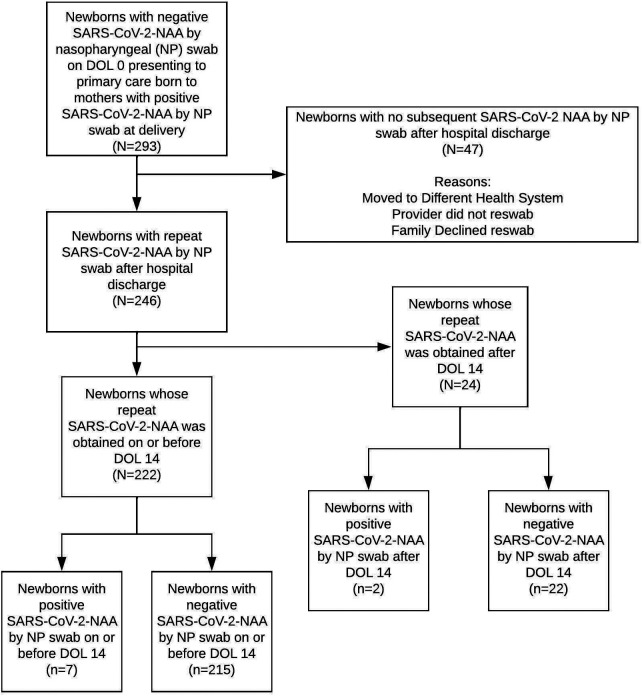
Flow chart of newborns born to women with a positive NAA test for SARS-Cov-2.

There were 293 infants born to SARS-CoV-2-positive mothers evaluated at CCMC practices between 27 March 2020 and 18 March 2022 (Figure 1). Of those, 222 newborns had repeat swab by DOL 14 and were included in the analysis (Table 1). The mean maternal age was 29.5 years; 29.3% of mothers were symptomatic for SARS-CoV-2 at delivery. The mean newborn gestational age was 38.5 weeks; 57.8% were male; 76.1% were non-White; 69.5% had Medicaid or were uninsured; and 29.8% were admitted to the NICU for reasons unrelated to maternal SARS-CoV-2 infection.

All infants tested negative for SARS-CoV-2 at DOL 0. The second SARS-CoV-2 test was obtained between DOL 2 and DOL 14 (median DOL 10). Among the second round of tests, 154 (69.4%) occurred when the alpha variant was the predominant strain, 58 (26.1%) during the delta strain, and 10 (4.5%) during the omicron strain. Of those, seven tested positive (3.2%) and none had symptoms attributable to SARS-CoV-2 infection. Details of the cases are included in Supplementary Table S1. No neonate in the cohort required emergency room care or hospital care by DOL 28.

## Discussion

We present the largest series of neonates born to SARS-CoV-2-positive mothers followed across a regional primary care network with many racial/ethnic minorities and Medicaid/uninsured populations. Vertical transmission is thought to occur through three primary mechanisms: *in utero* transmission through binding of viral spike proteins to receptors on host cell membranes; intrapartum transmission, which occurs during the process of labor and childbirth; and postnatal transmission, when the infant is exposed to maternal breast milk and/or respiratory secretions (9, 10). In this observational cohort, the rate of congenital infection as evidenced by a positive test at 24 h of age was zero; the perinatal transmission rate was 3.2% by DOL 14. These findings are similar to those in existing published cohorts: one prospective observational study found that of 177 newborns exposed to COVID-19, only 9 (5.1%) tested positive for the virus within 14 days of life via nasopharyngeal reverse transcription polymerase chain reaction (11). Similarly, previous studies have shown that the overall reported transmission rates were less than 5% (4–8, 12). Another key finding in this study is that the perinatal transmission rate persisted across multiple predominant strains in the New York metropolitan region, which mirrored that of the United States and abroad (6). Low transmission rates of similar hospital rooming-in and breastfeeding practices suggest post-hospitalization transmission mechanisms (5, 13).

The low overall transmission rates and the absence of symptomatic infection support the safety of the November 2022 AAP guidelines (3) recommending that infants born to asymptomatic mothers be allowed to room-in and directly breastfeed with appropriate masking and hand and breast hygiene. Our findings also support the safety of direct breastfeeding after hospital discharge with appropriate hand and breast hygiene. This study is limited by the short follow-up period and parent-reported adherence to hygiene and isolation practices. In addition, since the study was conducted in a single hospital system, it may not apply to other populations. However, our study represents a more regional and population-based estimate of perinatal SARS-CoV-2 transmission than prior reports.

## Data Availability

The raw, de-identified data supporting the conclusions of this article will be made available by the authors, without undue reservation. Requests to access the datasets should be directed to SJ, sjan1@northwell.edu.
