# Supplementary material for: Prevalence of SARS-CoV-2 in newborns born to SARS-CoV-2-positive mothers at 2 weeks of life
Source: Front Pediatr. 2024 Apr 25;12:1381104. doi: 10.3389/fped.2024.1381104 (PMC11079207; doi:10.3389/fped.2024.1381104)
Supplement: Supplementary file 1 [file Table1.docx]

**Supplementary Table 1. Case Details of Newborns to SARS-CoV-2 positive mothers**

| Case | SARS-Co-V-2 positive | Clinical Description |
| --- | --- | --- |
| 1 | DOL 6 | Infant was male born at 34 weeks gestation by emergency cesarean section due to impending respiratory failure of his mother, who was admitted several days earlier for respiratory distress secondary to SARS-Co-V-2. Mother was gravida 5 para 3. She is of Hispanic descent. APGARS at delivery were 8 and 9 at 1 and 5 minutes respectively. The newborn, who was separated from the mother immediately after birth, experienced respiratory distress at delivery, required resuscitation, and was transferred to a neonatal intensive care unit for management of prematurity, hypoglycemia, and difficulties with thermoregulation, and hyperbilirubinemia requiring phototherapy. Infant was initially SARS-Co-V-2 negative at 24 hours of life, but tested SARS-Co-V-2 positive at DOL 6. Father of the child was not tested. Child was followed clinically, and remained asymptomatic. Repeat SARS-Co-V-2 testing at DOL 14 and DOL 28 were negative. At 9 months of age, the infant remains healthy and is meeting all developmental milestones. |
| 2 | 25 | Infant was a male born at term by cesarean section for prior cesarean section to a mother who was gravida 1 para 0 and asymptomatic during the period leading up to and including delivery. Maternal history was significant for an incompletely treated urinary tract infection in the third trimester. Mother is of African-American descent. After discharge, the mother remained asymptomatic and wore a mask at home. The infant was exclusively fed by his mother, both breastfeeding and feeding with formula. Diapers were changed exclusively by his mother. The infant lived with both parents. Child was not tested before DOL 14; repeat testing on DOL 25 was positive. Follow-up testing on DOL 50 was negative. Child was remained asymptomatic for SARS-Co-V-2. At 9 months of age, the infant remains healthy and is meeting all developmental milestones. |
| 3 | 12 | Infant was male born at 36 weeks gestation by cesarean section for repeat cesarean and twin gestation to a 33 year old mother who was gravida 7 para 3 and was asymptomatic for SARS-Co-V-2 infection. Mother is of African American descent. Maternal history was significant for gestational hypertension and diet-controlled gestational diabetes. Nursery course was complicated by hypoglycemia and abdominal distension which resolved prior to discharge. The infant was fed by direct breastfeeding, expressed breast milk, and formula while in the hospital and at home; he was discharged at 3 days of life. While at home, mother remained masked during the isolation period. The infant and his twin brother were fed and had their diapers changed by both his mother and father. Mother remained masked at home during the observation period. Infant was SARS-Co-V-2 positive on DOL 12. Notably, the infant’s twin brother was SARS-Co-V-2 negative on DOL 12. Both twins remained asymptomatic for SARS-Co-V-2 infection. At 9 months of age, the infant remains healthy and is meeting all developmental milestones. |
