# Supplementary material for: Prevalence of SARS-CoV-2 in newborns born to SARS-CoV-2-positive mothers at 2 weeks of life
Source: Front Pediatr. 2024 Apr 25;12:1381104. doi: 10.3389/fped.2024.1381104 (PMC11079207; doi:10.3389/fped.2024.1381104)
Supplement: Supplementary file 2 [file Table2.docx]

**Supplementary Table 2: Characteristics of Newborns born to SARS-CoV-2 Positive Mothers, Mothers, Delivery Circumstances, and Home Circumstances across Covid Waves**

| **Maternal Characteristics*** | **Alpha** | **Delta** | **Omicron** | **P Value** |
| --- | --- | --- | --- | --- |
| Mean age (range) | 29.3 + 6.2 | 30.5 + 5.1 | 27.8 + 6.7 | 0.287 |
| Gravid mean (range) | 2.4 + 1.9 | 2.4 + 1.4 | 2.2 + 1.2 | 0.917 |
| Parity mean (range) | 1.1 + 1.2 | 0.9 + 1.2 | 0.6 + 0.7 | 0.297 |
| Ever symptomatic for COVID-19 |  |  |  |  |
| Yes, n (%) | 38 (27.1) | 16 (35.6) | 2 (33.3) | 0.546 |
| No, n (%) | 102 (72.9) | 29 (64.4) | 4 (66.7) |  |
|  |  |  |  |  |
| **Newborn Characteristics**** | **Alpha** | **Delta** | **Omicron** | **P Value** |
| Gestational Age at birth in weeks, mean (range) | 38.5 + 1.7 | 38.6 + 1.4 | 38.0 + 1.6 | 0.611 |
| Sex |  |  |  |  |
| Female, N (%) | 67 (43.5) | 25 (43.1) | 2 (20.0) | 0.342 |
| Male, N (%) | 87 (56.5) | 33 (56.9) | 8 (80.0) |  |
| Race |  |  |  |  |
| White | 44 (29.9) | 7 (12.5) | 2 (20.0) | 0.022 |
| Black/African American | 49 (33.3) | 15 (26.8) | 3 (30.0) |  |
| Asian | 14 (9.5) | 15 (26.8) | 2 (20.0) |  |
| Other | 40 (27.2) | 19 (33.9) | 3 (30.0) |  |
| Ethnicity |  |  |  |  |
| Hispanic | 32 (24.2) | 7 (28.0) | 2 (28.6) | 0.901 |
| Non-Hispanic | 100 (75.8) | 18 (72.0) | 5 (28.6) |  |
| Insurance |  |  |  |  |
| Commercial, N (%) | 49 (31.8) | 16 (30.2) | 3 (30.0) | 0.383 |
| Medicaid, N(%) | 99 (64.3) | 31 (58.5) | 6 (60.0) |  |
| Uninsured, N (%) | 6 (3.9) | 6 (11.3) | 1 (10.0) |  |
| Delivery Method |  |  |  |  |
| Vaginal, n (%) | 110 (71.4) | 37 (63.8) | 9 (90.0) | 0.209 |
| Cesarean Section, n(%) | 44 (28.6) | 21 (36.2) | 1 (10.0) |  |
| Birthweight in kilograms, mean (range) | 3.1 + 0.5 | 3.2 + 0.5 | 3.0 + 0.5 | 0.308 |
|  |  |  |  |  |
| **Delivery Characteristics***** |  |  |  |  |
| Birth Hospital | **Alpha** | **Delta** | **Omicron** | **P Value** |
| Long Island Jewish | 95 (64.6) | 39 (67.2) | 6 (60.0) | 0.081 |
| North Shore University Hospital | 22 (15.0) | 13 (22.4) | 1 (10.0) |  |
| South Shore University Hospital | 10 (6.8) | 1 (1.7) | 2 (20.0) |  |
| Lenox Hill | 4 (2.7) | 0 | 1 (10.0) |  |
| Forest Hills | 0 | 2 (3.4) | 0 |  |
| Huntington Hospital | 2 (1.4) | 1 (1.7) | 0 |  |
| Non-Northwell Hospitals**** | 14 (9.5) | 2 (3.4) | 0 |  |
| AGPAR |  |  |  |  |
| 1 Minute | 8.4 + 0.8 | 8.2 + 1.5 | 8.5 + 1.0 | 0.365 |
| 5 Minute | 8.9 + 0.4 | 8.7 + 1.0 | 8.9 + 0.3 | 0.325 |
| Hospital Course |  |  |  |  |
| Well baby nursery | 59 (70.2) | 59 (100) | 0 | .086 |
| NICU | 25 (29.8) | 23 (92.0) | 2 (8.0) |  |
| Discharged by 24 hours |  |  |  |  |
| Yes | 69 (46.0) | 14 (24.1) | 4 (44.4) | 0.015 |
| No | 81 (54.0) | 44 (75.9) | 5 (55.6) |  |
| Precautions Taken at Home by DOL 14***** |  |  |  |  |
| Direct breastfeeding | 84 (54.5) | 30 (51.7) | 8 (80.0) | 0.248 |
| Expressed breast milk | 38 (24.7) | 12 (20.7) | 2 (20.0) | 0.802 |
| Formula | 134 (87.0) | 45 (77.6) | 5 (50.0) | 0.005 |
| *Age, Gravidy: n=220; Parity: n=215.  **Gestational age: n=220; Race: n=213; Ethnicity: n=164; Insurance: n=217.  ***APGAR: N=213; Discharged by 24 hours: n=217  ****Non-Northwell Health Hospitals included Good Sam, St. Charles, St. Catherine’s, Mt. Sinai, and Mercy  *****Babies could have used a combination of feeding modalities | | | | |
